# Impact of Sepsis Management Guideline Implementation in Emergency Departments: A Systematic Review of Process Measures and Clinical Outcomes

**DOI:** 10.7759/cureus.108271

**Published:** 2026-05-04

**Authors:** Mohammed A Khormi, Nada M Alshehri, Nada F Albishri, Abdulelah Y Beati, Khadijah O Mashraqi, Fahad M Nasser, Rawan F Alharbi, Abdulaziz A Almousa, Nawaf M Alqahtani, Khalid F Salaemae, Mohmad H Al Subaie

**Affiliations:** 1 Family Medicine, Eskan Alrawan PHC, Jazan Health Cluster, Jazan, SAU; 2 College of Medicine, King Khalid University, Abha, SAU; 3 College of Medicine, Jazan University, Jazan, SAU; 4 College of Medicine, Arabian Gulf University, Manama, BHR; 5 College of Medicine, King Abdulaziz University, Jeddah, SAU; 6 College of Medicine, King Faisal University, Al Ahsa, SAU; 7 College of Medicine, Prince Sattam bin Abdulaziz University, Al Kharj, SAU; 8 College of Medicine, Umm Al-Qura University, Makkah, SAU; 9 Faculty of Medicine, King Faisal University, Hofuf, SAU

**Keywords:** antimicrobial therapy, clinical outcomes, emergency department, guideline implementation, lactate measurement, sepsis, sepsis bundles, sepsis guidelines, surviving sepsis campaign, time to antibiotics

## Abstract

Sepsis is a life-threatening condition associated with significant morbidity and mortality worldwide. The emergency department (ED) plays a crucial role in the early recognition and management of sepsis, as delays in treatment are strongly associated with worse outcomes. Although sepsis management guidelines and care bundles have been widely implemented to standardize and accelerate care, their impact on patient outcomes remains variably reported. This systematic review evaluated the effect of implementing sepsis management guidelines in EDs on both process indicators and clinical outcomes. A systematic search was conducted across PubMed, Scopus, Web of Science, and the Cochrane Library using a combination of controlled vocabulary and free-text terms related to sepsis, emergency care, and guideline implementation, yielding 4,309 records; after removal of duplicates and screening, eight studies met the inclusion criteria. Data extraction was performed using a standardized form capturing study characteristics, interventions, and outcomes, and risk of bias was assessed using the Risk Of Bias In Non-randomized Studies of Interventions (ROBINS-I) tool. The included studies comprised diverse designs, including before-and-after studies, retrospective cohort studies, observational studies, and one quasi-experimental trial. Overall, implementation of sepsis guidelines consistently improved process measures, including reduced time to antibiotic administration, increased lactate testing, improved blood culture collection, and higher compliance with sepsis bundles. However, effects on clinical outcomes were heterogeneous: some studies reported reductions in mortality, intensive care unit (ICU) admissions, and resource utilization, while others showed no significant mortality benefit despite improved process indicators. Additionally, some studies reported unintended consequences such as increased antimicrobial use and potential overtreatment. Most studies were judged to have a moderate to serious risk of bias, largely due to confounding and non-randomized designs. In conclusion, implementation of sepsis management guidelines in EDs is associated with consistent improvements in care processes and timeliness of treatment, but evidence for improved clinical outcomes, particularly mortality, remains inconsistent and is limited by study design and risk of bias; further high-quality studies are needed to establish causality, optimize implementation strategies, and balance timely treatment with antimicrobial stewardship.

## Introduction and background

Sepsis is a life-threatening organ dysfunction caused by a dysregulated host response to infection and is a major global contributor to morbidity and mortality [1,2]. It accounts for millions of deaths annually and remains a leading cause of preventable critical illness, particularly in low- and middle-income countries. Early clinical presentation is often nonspecific, which contributes to delayed recognition and treatment [3,4].

Emergency departments (EDs) are a key point of entry for patients with sepsis and septic shock, and the quality and speed of initial care strongly influence outcomes. Delays in antimicrobial therapy, fluid resuscitation, and source control are consistently associated with higher mortality, especially in septic shock [1-7]. However, delivering timely, evidence-based care in EDs is challenging due to crowding, time pressure, and limited diagnostic certainty.

To improve early management, several organizations have developed sepsis guidelines emphasizing rapid recognition and standardized treatment. The Surviving Sepsis Campaign (SSC) provides internationally endorsed recommendations, including time-sensitive bundles such as lactate measurement, blood culture collection before antibiotics, early administration of broad-spectrum antimicrobials, prompt fluid resuscitation, and ongoing hemodynamic assessment [3,5,7]. The World Health Organization (WHO) has also identified sepsis as a global health priority and supports integrated care strategies across the continuum from early recognition to definitive management. In addition, national bodies such as the Canadian Association of Emergency Physicians (CAEP) have adapted these recommendations for emergency care settings [1,4,8].

Implementation of sepsis guidelines in EDs often involves multifaceted interventions, including standardized protocols, triage screening tools, nurse-initiated order sets, and electronic alert systems [1-8]. These strategies have generally improved adherence to evidence-based processes of care, including faster antibiotic administration, increased lactate testing, and improved bundle compliance. However, the effect of these interventions on patient-centered outcomes remains inconsistent. While some studies report reductions in mortality, intensive care unit (ICU) admissions, and hospital length of stay, others show no significant mortality benefit after adjusting for illness severity. Concerns have also been raised regarding potential unintended effects, such as increased antimicrobial use, overtreatment of low-risk patients, and difficulties maintaining sustained compliance in busy ED environments. Therefore, a systematic synthesis of the evidence is needed to clarify the impact of ED-based sepsis guideline implementation on both process measures and clinical outcomes.

## Review

Methods

Search Strategy

A comprehensive electronic search was conducted in four databases: PubMed, Scopus, Web of Science, and the Cochrane Library. The search strategy combined controlled vocabulary and free-text terms related to sepsis, septic shock, ED care, and guideline implementation (Table 1).

**Table 1:** Search strategy used across databases This table summarizes the electronic search strategy applied in PubMed, Scopus, Web of Science, and the Cochrane Library for the systematic review. Searches combined controlled vocabulary (e.g., MeSH terms) and free-text keywords related to sepsis, septic shock, emergency department care, and guideline implementation. No date, language, or publication-type restrictions were applied. Duplicate records were removed prior to screening.

Database	Search terms / strategy
PubMed	("Sepsis"[MeSH] OR sepsis OR "septic shock") AND ("Emergency Service, Hospital"[MeSH] OR emergency department OR ED) AND (guideline OR protocol OR bundle OR "clinical protocol" OR implementation OR adherence)
Scopus	TITLE-ABS-KEY (sepsis OR "septic shock") AND TITLE-ABS-KEY ("emergency department" OR ED) AND TITLE-ABS-KEY (guideline OR bundle OR protocol OR implementation OR compliance)
Web of Science	TS=(sepsis OR "septic shock") AND TS=("emergency department" OR ED) AND TS=(guideline OR bundle OR protocol OR implementation OR adherence)
Cochrane Library	sepsis AND emergency AND (guideline OR protocol OR bundle OR implementation)

In PubMed, Medical Subject Headings (MeSH) and keywords were used, including “Sepsis,” “Septic Shock,” “Emergency Service, Hospital,” “Clinical Protocols,” and “Guideline Adherence,” along with implementation-related terms such as “compliance,” “adherence,” and “quality improvement.” Equivalent search strategies were adapted for Scopus (TITLE-ABS-KEY), Web of Science (TS), and the Cochrane Library to ensure consistency across databases.

Study Selection

Study selection followed the Preferred Reporting Items for Systematic Reviews and Meta-Analyses (PRISMA) 2020 guidelines [9]. After removal of duplicates, 2,809 records were screened by title and abstract, and 2,650 were excluded due to irrelevance.

Full-text review was conducted for 159 articles. Of these, 151 were excluded for the following reasons: no evaluation of implementation strategies (n=60), not conducted in ED settings (n=40), absence of relevant outcomes (n=30), and study design limited to reviews or editorials (n=21).

Ultimately, eight studies met the inclusion criteria and were included in the qualitative synthesis. Disagreements during screening were resolved through discussion and consensus.

Data Extraction

Data were extracted using a standardized form capturing study characteristics, including author, year, country, study design, sample size, population, intervention, comparator, and outcomes.

Process outcomes included time to antibiotic administration, lactate measurement, blood culture collection, fluid resuscitation, and sepsis bundle compliance. Clinical outcomes included mortality, ICU admission, hospital length of stay, and other patient-centered outcomes.

Risk of Bias Assessment

Risk of bias was assessed using the Risk Of Bias In Non-randomized Studies of Interventions (ROBINS-I) tool [10]. Seven domains were evaluated: confounding, participant selection, classification of interventions, deviations from intended interventions, missing data, outcome measurement, and selection of reported results.

Each domain was rated as low, moderate, or serious risk of bias, and an overall judgment was assigned based on the highest level of risk identified in any domain.

Results

Study Selection

**Figure 1:**
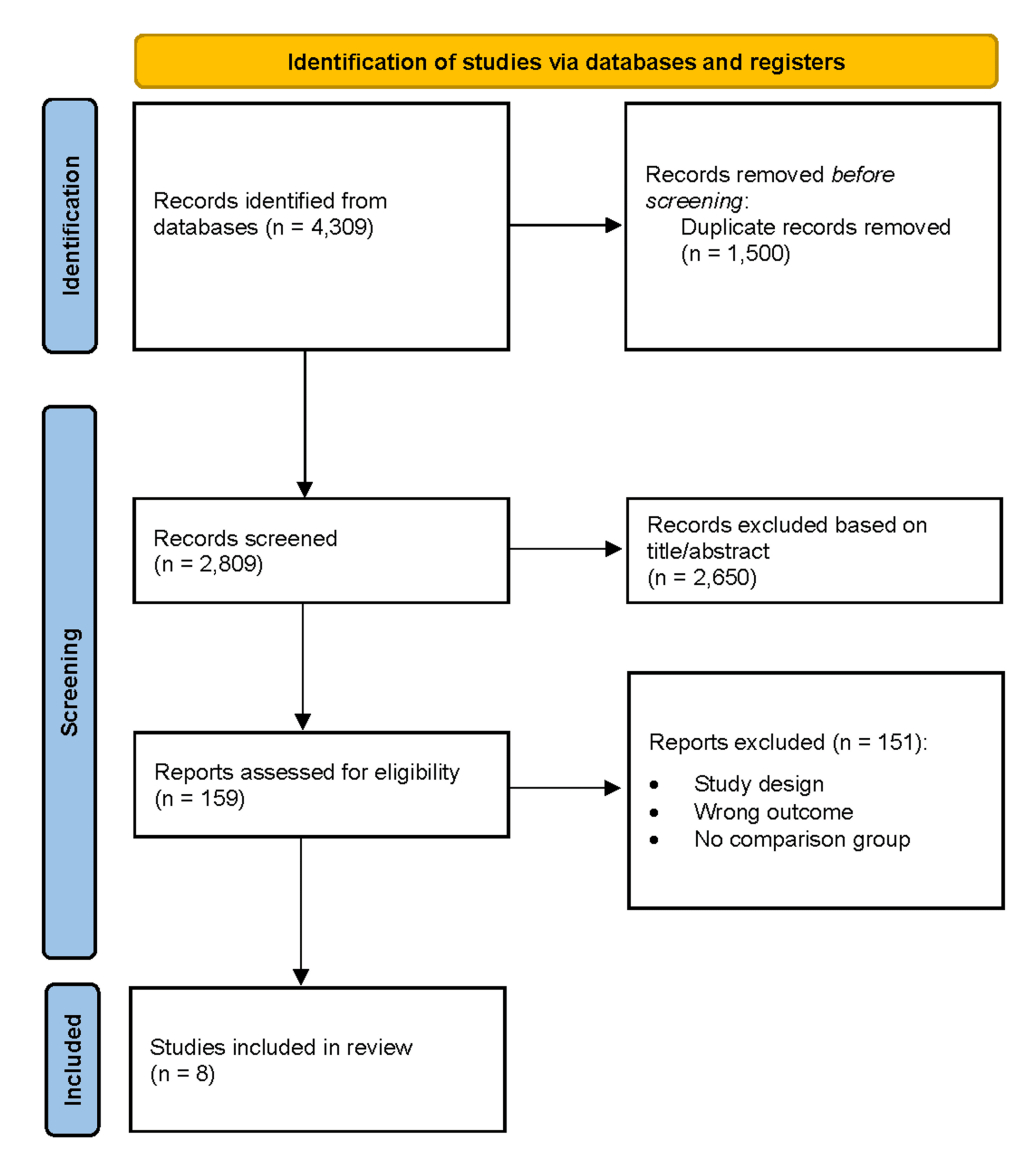
PRISMA flow diagram depicting the study selection process for the systematic review Preferred Reporting Items for Systematic Reviews and Meta-Analyses (PRISMA) flow diagram detailing the study selection process for this systematic review. Following the identification of records through database searching and other sources, duplicates were removed, and the remaining records were screened. Full-text articles were assessed for eligibility, with exclusions documented along with reasons. Studies meeting the inclusion criteria were included in the final synthesis.

The database search yielded 4,309 records. After removal of duplicates (n = 1,500), 2,809 records were screened by title and abstract, and 2,650 were excluded. A total of 159 full-text articles were assessed for eligibility, of which 151 were excluded due to predefined criteria. Eight studies were included in the final qualitative synthesis (Figure 1) [1-8].

Study Characteristics

The eight included studies were conducted in multiple countries, including the United States, Canada, Australia, Italy, and Denmark. Study designs included before-and-after studies, retrospective cohorts, prospective observational studies, and one quasi-experimental pragmatic trial. Sample sizes ranged from 104 to over 49,000 participants. Most studies included adult patients presenting to the ED with sepsis or severe infection, defined using Sepsis-2, Sepsis-3, or systemic inflammatory response syndrome (SIRS) criteria (Table 2).

**Table 2:** Characteristics and outcomes of studies evaluating the impact of sepsis guideline implementation in emergency departments This table summarizes the key characteristics, interventions, and outcomes of studies included in the systematic review assessing the impact of sepsis guideline implementation in emergency department (ED) settings. Studies are organized by first author and reference number. For each study, the country, study design, sample size, patient population, intervention strategy, comparator group, and reported outcomes are presented. Outcomes are categorized into process measures (e.g., time to antibiotics, lactate measurement, blood culture collection, fluid resuscitation, and sepsis bundle compliance) and clinical outcomes (e.g., mortality, intensive care unit [ICU] admission, hospital length of stay, and cost). Key findings are summarized to highlight the direction and magnitude of effects reported in each study. Abbreviations: AMS, antimicrobial stewardship; ATS, American Thoracic Society; CAEP, Canadian Association of Emergency Physicians; CI, confidence interval; DART, Detection and Response Team; DiD, difference-in-differences; ED, emergency department; ICU, intensive care unit; ID, infectious diseases; ITS, interrupted time series; LOS, length of stay; NEWS-2, National Early Warning Score 2; NSW, New South Wales; OR, odds ratio; QI, quality improvement; RN, registered nurse; SIRS, systemic inflammatory response syndrome; SSC, Surviving Sepsis Campaign; WHO, World Health Organization.

Study	Country	Design	Sample	Population	Intervention	Comparator	Outcomes (Process)	Outcomes (Clinical)	Key Findings
Seymour [1]	USA	Retrospective cohort (statewide mandated protocol)	49331	ED severe sepsis & septic shock (Sepsis-2)	State-mandated sepsis protocols (3h & 6h bundles)	Time to treatment variation	Time to bundle, antibiotics, fluids	In-hospital mortality	Treatment delays associated with increased mortality (OR 1.04/hour); antibiotic delay increased mortality; fluid timing not significant
Peltan [2]	USA	Pragmatic clinical trial (quasi-experimental, DiD)	10,151 sepsis patients	ED sepsis (Sepsis-3)	Code Sepsis protocol (team-based rapid response workflow)	Pre vs post + control hospitals	Door-to-antibiotics, bundle timing	30-day mortality, hospital mortality, LOS, safety outcomes	Antibiotic time reduced ~13 min (p<0.001); increased antibiotic use; mortality unchanged (aOR 0.90)
McColl [3]	Canada	Before–after	352 (167 pre, 185 post)	ED severe infection (SIRS-based)	Sepsis bundle + system redesign (triage flagging, RN directive, education, protocol)	Pre vs post	Time to MD review, fluids, antibiotics, protocol use	30-day mortality, ICU admission, vasopressors	Mortality decreased 30.7%→17.3% (p=0.006); protocol use increased 20%→80%; faster antibiotics and fluids; ICU admission decreased
Moore [4]	USA	Before–after (QI project)	181 charts (90 pre, 91 post)	ED sepsis patients	Nurse-driven DART protocol + checklist tool	Pre vs post	Lactate, blood cultures, antibiotics, bundle compliance, time to screen	ED LOS, hospital days, cost	Bundle compliance increased 30%→80%; improved lactate, cultures, antibiotics; hospital length of stay reduced ~2.5 days
Plambech [5]	Denmark	Prospective interventional (audit-based)	104 sepsis patients	ED sepsis (SIRS-based)	Sepsis guideline implementation (training + posters + checklists + audit feedback)	Baseline vs 18 weeks vs 1 year	Lactate, antibiotics, blood culture, fluids, adherence	Not reported	Adherence increased 37%→65% at 18 weeks (p=0.03), declined at 1 year; improved antibiotics and cultures
Corsini [6]	Italy	Before–after (retrospective + ITS)	1079 (577 pre, 502 post)	ED sepsis (Sepsis-3)	AMS program (training + audit + feedback + NEWS-2 integration + ID support)	Pre vs post	Sepsis recognition, blood cultures, antibiotics, fluids, bundle compliance	14-day mortality	Recognition increased 47.5%→61%; antibiotics 25.7%→46.8%; bundle compliance OR 2.99; guideline adherence OR 4.54; improved survival association
Leisman [7]	USA	Prospective multicenter observational (3 cohorts)	~14,755 total	Severe sepsis & septic shock (SIRS + organ dysfunction)	3-hour sepsis bundle compliance	Compliant vs non-compliant	Time-based bundle adherence (antibiotics, fluids, lactate, cultures)	In-hospital mortality, cost	Mortality decreased across cohorts (OR 0.60–0.84); cost reduction; consistent survival benefit
Romero [8]	Australia	Pre–post retrospective audit	329 (164 pre, 165 post)	ED adult sepsis patients	NSW sepsis guideline implementation (education + triage tool + protocol)	Pre vs post	Time to antibiotics, triage category, lactate testing, IV fluids	ICU admission, ED flow metrics	Time to antibiotics reduced 230 min; lactate testing increased 36.6%→67.9%; more urgent triage; ICU admission decreased significantly

Interventions primarily involved implementation of sepsis management protocols or bundles, including structured pathways, nurse-driven protocols, antimicrobial stewardship (AMS) programs, and system-level mandated care pathways. Implementation strategies commonly included staff education, audit and feedback, triage screening tools, and multidisciplinary coordination. Comparators were typically pre-implementation versus post-implementation periods, although some studies used compliant versus non-compliant groups or difference-in-differences designs.

Risk of Bias Within Studies

Overall, the included studies showed predominantly moderate to serious risk of bias. Confounding was the most frequent limitation due to non-randomized and observational designs. Selection bias was generally moderate, reflecting retrospective data sources and reliance on diagnostic coding. Classification of interventions was mostly low risk due to clearly defined protocols (Table 3).

**Table 3:** Risk of bias assessment of included studies using the ROBINS-I tool This table presents the risk of bias assessment for each included study in the systematic review using the Risk Of Bias In Non-randomized Studies of Interventions (ROBINS-I) tool [10]. Each study is evaluated across seven domains: confounding, selection of participants, classification of interventions, deviations from intended interventions, missing data, outcome measurement, and reporting bias. An overall risk of bias judgment is provided for each study based on the highest level of risk identified across domains. Ratings are categorized as low, moderate, or serious risk of bias. Most studies included in this review were observational or quasi-experimental in design, and therefore inherently subject to potential confounding and selection bias. The assessment highlights variability in methodological quality across studies, with confounding and deviations from intended interventions representing the most common sources of bias.

Study	Confounding	Selection	Intervention Classification	Deviations from Intervention	Missing Data	Outcome Measurement	Reporting Bias	Overall Risk
Seymour [1]	Serious	Low	Low	Moderate	Low	Low	Moderate	Serious
Peltan [2]	Moderate	Low	Low	Moderate	Low	Low	Moderate	Moderate
McColl [3]	Serious	Moderate	Low	Moderate	Low	Low	Moderate	Serious
Moore [4]	Serious	Moderate	Low	Serious	Moderate	Moderate	Moderate	Serious
Plambech [5]	Serious	Moderate	Low	Serious	Moderate	Moderate	Moderate	Serious
Corsini [6]	Serious	Moderate	Low	Moderate	Moderate	Low	Moderate	Serious
Leisman [7]	Serious	Moderate	Low	Moderate	Low	Low	Moderate	Serious
Romero [8]	Serious	Moderate	Low	Serious	Moderate	Moderate	Moderate	Serious

Bias due to deviations from intended interventions was frequently moderate to serious, particularly in quality improvement studies with variable adherence. Missing data was generally low to moderate. Outcome measurement was mostly low risk due to objective endpoints such as mortality and time-based measures. Reporting bias was moderate across most studies due to multiple outcomes and exploratory analyses. Overall, most studies were rated as having a serious risk of bias, with only one study rated as moderate.

Process Outcomes

Across studies, implementation of sepsis guidelines consistently improved ED process measures. Improvements included reduced time to antibiotic administration, increased lactate testing, improved blood culture collection, enhanced fluid resuscitation, and higher sepsis bundle compliance. Reported reductions in time to antibiotics ranged from over 200 minutes in one study to approximately 10-30 minutes in others. Bundle compliance also increased substantially, in some cases rising from approximately 30% to 80% following protocol implementation.

Clinical Outcomes

Clinical outcomes were more variable. Several studies reported reductions in mortality and ICU admission rates following implementation of sepsis guidelines, while others showed no statistically significant mortality difference despite improved process measures. Large observational data suggested an association between full bundle compliance and reduced mortality, whereas at least one quasi-experimental study found no significant mortality benefit despite faster treatment delivery.

Qualitative Synthesis of Findings

Across studies, four main themes emerged: improved process efficiency, faster time to treatment, variable effects on clinical outcomes, and unintended consequences. Improvements in ED workflow and triage performance were consistently reported, with increased protocol use, faster initial assessment, and higher adherence to structured sepsis pathways.

Time to treatment improved across most studies, particularly for antibiotic administration and fluid resuscitation, with several reporting clinically meaningful reductions in door-to-antibiotic time. Mortality findings were inconsistent. Some studies demonstrated reduced mortality and ICU admission rates associated with higher bundle compliance, while others showed no significant difference despite improved process metrics.

Unintended effects included increased antimicrobial exposure, potential overtreatment of non-infected patients, and challenges in sustaining long-term adherence. Some studies also reported declines in compliance over time and highlighted workflow strain on ED staff due to time-sensitive bundle requirements.

Limitations

The findings of this review should be interpreted in light of several limitations. Most included studies were non-randomized and observational in design, introducing a moderate to serious risk of bias, particularly from confounding and reliance on retrospective data. Heterogeneity in study designs, interventions, and outcome measures further limits comparability across studies. In addition, variability in sepsis definitions, including the use of both Sepsis-2 and Sepsis-3 criteria, likely contributed to differences in reported outcomes and reduced overall consistency in the evidence base.

## Conclusions

Sepsis management guidelines in the ED improve the timeliness and consistency of care delivery by standardizing key diagnostic and treatment processes. However, while these protocols enhance early recognition and treatment speed, their impact on clinical outcomes is not uniform, and careful attention is needed to avoid unnecessary treatment in patients without true infection. Future research should focus on strengthening study design to better evaluate causality, improving diagnostic precision to support appropriate treatment decisions, and developing implementation strategies that are sustainable and practical in busy ED settings.
